# Research on influencing factors of public panic during infectious disease pandemics: a structural equation modeling study in five cities

**DOI:** 10.3389/fpubh.2026.1834066

**Published:** 2026-05-15

**Authors:** Fang Liu, Yi Bao Wang, Wendong Xu

**Affiliations:** Emergency Management Department, University of Mining and Technology, Xuzhou, China

**Keywords:** emergency management, infectious disease transmission, influencing factors, public health policy, public panic, structural equation modeling

## Abstract

Infectious diseases continue to pose a persistent threat to global public health, drawing greater focus to their transmission dynamics—especially the processes through which these dynamics induce public panic. While this aspect is vital to successful epidemic control, it has not been adequately theorized in prior research. To fill this gap, questionnaire data from 660 residents across five Chinese cities (Nanjing, Xuzhou, Suzhou, Shanghai, and Lianyungang) were examined via structural equation modeling (SEM) using IBM SPSS Amos 26.0. The analysis identified five key dimensions shaping public panic during respiratory infection outbreaks: personal characteristics, event perception, information spread, assessments of preventive interventions, and collective conduct. The measurement model demonstrated sound psychometric properties, with all factor loadings exceeding 0.50 and acceptable reliability (Cronbach's α ranging from 0.68 to 0.89). High loadings were observed for information transparency (0.794), government material allocation (0.854), and community prevention (0.834), indicating that these observed variables strongly represent their respective latent constructs. Event perception showed notable associations with information factors and government evaluations (correlations 0.70–0.77), highlighting the interconnected nature of these determinants. These results carry direct policy relevance. Upholding information accuracy, combating misinformation, and improving public grasp of disease transmission processes are theoretically and ethically sound strategies for curbing public panic, as they directly address the core constructs identified in the measurement model. Ensuring information transparency and delivering consistent risk communication can interrupt herd behavior cascades during health crises. The research offers practical guidance for establishing evidence-based emergency management procedures and public health preparedness frameworks, emphasizing the central role of these determinants in designing effective risk communication and emergency management policies.

## 1 Introduction

Infectious disease outbreaks continue to present an intimidating challenge to global public health security. Historical cases—spanning from the 1918 influenza pandemic to 21st-century epidemics such as SARS, H1N1, Ebola, and most recently COVID-19—demonstrate pathogens' capacity to quickly strain healthcare systems, undermine economic stability, and disrupt society's core fabric. In an ever-more interconnected global context, this threat is not merely biomedical but also profoundly psychosocial, characterized by transmission dynamics that function not only in biological carriers but also within the complex ecosystems of human information and behavior (1).

Public panic—defined here as an acute, often disproportionate fear and anxiety reaction that can prompt irrational and potentially maladaptive behaviors—is a critical yet frequently overlooked variable in epidemic containment (2). Theoretically, panic can be understood through established frameworks such as the Protection Motivation Theory (3) and the Extended Parallel Process Model (4), which posit that perceived threat severity, vulnerability, and self-efficacy jointly determine emotional and behavioral responses to health risks. As Fast et al. demonstrated, social responses can be framed as a contagion process in their own right, propagating through populations via local interactions and media signals; the spread of panic, in turn, relies on disease prevalence, perceived risk, and global media exposure (5). As demonstrated by Li et al., public panic during the early stage of COVID-19 in China exhibited distinct patterns modulated by stringent prevention measures (6). These behaviors—ranging from hoarding essential supplies and shunning public spaces to stigmatizing specific groups and losing trust in public health authorities—can notably exacerbate the crisis (7). They can impede the effective implementation of non-pharmaceutical interventions, disrupt supply chains, and divert critical resources from affected regions, thereby amplifying the very threat people aim to avoid (8). Understanding these dynamics is essential, as disaster research has repeatedly challenged the “panic myth”—the belief that the public will inevitably act irrationally during crises—revealing that prosocial behavior and orderliness are far more common than unbridled chaos (2).

From a psychological standpoint, panic represents an interconnected behavioral and emotional reaction to perceived threats or danger. Risk perception theory suggests that individuals' subjective judgments about the probability and severity of harm are central to shaping emotional arousal (9). In public health emergency scenarios, the sudden and often unforeseeable nature of these crises leaves individuals with insufficient time to adapt psychologically. This vulnerability is further heightened by the general public's limited understanding of medical knowledge, which impairs their capacity to maintain emotional balance when confronting rapidly deteriorating and severe outbreaks. Such emotional fragility can spill over into widespread panic, ultimately jeopardizing both individual mental health and overall social stability (10). Lee et al. (11) found that the public‘s risk perception increased state anxiety, which in turn deepened their willingness to engage in herding behavior. The COVID-19 pandemic clearly illustrated how ambiguity surrounding virus transmission mechanisms can amplify collective anxiety, as uncertainty about infection pathways and preventive measures created a fertile environment for psychological distress (12, 13).

Effective emergency management amid public health crises hinges not only on scientifically sound policymaking at the governmental level but also on a nuanced understanding of public behavioral patterns (14). Wu and Li (15) demonstrated that individuals who perceived experts' advice as useful and shared risk information with friends reported lower risk perception. When a major public health crisis arises, ordinary citizens find themselves on the front lines—confronting the threat directly and, through their daily decisions, subtly influencing the course of the outbreak. Whether choosing to wear a mask, limit social gatherings, or come forward for vaccination, these individual actions, multiplied across millions of households, collectively determine the outcome of containment efforts (16). As such, the effectiveness of public health measures depends not only on scientific rigor but also on the collaboration of entire communities. This places a dual responsibility on authorities: they must utilize technical expertise to halt viral transmission, while simultaneously addressing public anxieties, fostering constructive responses, and upholding societal stability. In essence, the battle is waged on two fronts—one against the pathogen itself, and another, equally vital, for public trust and social unity (17). Jin et al. (18) showed that a higher appraisal of risk communication mitigated susceptibility to emotional contagion during the early outbreak period.

This study employed survey data collected during the COVID-19 pandemic to enhance understanding of how psychological distress arises and persists in a crisis setting. The questionnaire was developed to capture a range of factors that may shape public responses, including personal demographics, individuals' interpretations of the health threat, the types of information they were exposed to, their views on official prevention measures, and the influence of their social circles. Structural equation modeling was subsequently utilized to trace how these varied factors contributed to public anxiety and behavioral reactions. The research objective is threefold: (1) to identify the key dimensions (event cognition, information factors, government factors, group factors, and personal characteristics) that influence public panic during infectious disease pandemics; (2) to empirically test the relative strength of these factors using structural equation modeling; and (3) to provide evidence-based recommendations for crisis communication and emergency management. The research objective went beyond simple event description; instead, it aimed to identify the underlying patterns that repeatedly emerge when fear spreads throughout a population. For policymakers and public health professionals, the study's findings are intended to deliver practical utility—providing clearer insights into the sources of public panic and guiding the creation of targeted messages and interventions that can reach individuals before fear escalates.

From the perspective of sociology, Qin and Zhou (19) deconstructed and studied people's negative psychological reactions in emergencies from the perspective of sociology, from the three dimensions of public right to know, social trust, and public management. Shan et al. (20) found that the public's behavior attitude, subjective norms and perceptual behavior control are the main factors affecting the public's perception of food additive safety risks and panic behavior.

From the existing literature, the influencing factors of public panic can be roughly divided into three categories. The first is the characteristics of emergencies and the cognition of emergencies. People's unpredictability and uncertainty about emergencies are closely related to panic (21, 22), and Armfield believes that people's sense of uncontrollability can also trigger panic about specific things (23). The second is the information factor. The uncertainty of risk exacerbates the thirst and anxiety of all segments of society for information. The public is highly vulnerable to the sources, perceptions and value acceptance of risk information (24), the public is quite sensitive to the acquisition of risk information, and the moderate dissemination of risk information makes the audience vigilant about some potential risks, which can improve the public's risk tolerance and alleviate social panic (25), media panic theory holds that media communication tends to exaggerate real threats, set unnecessary social panic agendas, affect public safety and its confidence, and lead to new and more panic phenomena or panic psychology (26), the third is the government's prevention and control factors. Panic in emergencies may also stem from distrust of government (20), in emergency management, the leadership and command of the government is crucial, and the government's crisis response and handling capabilities are closely related to the public's trust index in the government. Li et al. (27) revealed that effective risk communication enhances public trust in government, which in turn moderates public anxiety during health crises. The fundamental solution to prevent and resolve people's panic in emergencies is to establish a trust mechanism for the whole society, and to establish or restore a relationship of trust between people, people and the government, people and society, and people and the environment (28), which includes the establishment and improvement of crisis management mechanisms, social integration mechanisms and social security mechanisms. In addition, some scholars have proposed that strong social support such as the reserves of materials and medicines prepared by governments at all levels and various groups for disaster emergency relief, the setting up of evacuation sites, and psychological education are conducive to the public's appropriate self-help and alleviate the sense of fear in their hearts (29). The diversity of factors influencing public panic is evident, so there is a need for a multidimensional exploration of the structure of public panic. Reifegerste and Rossmann (30) identified distinct seasonal patterns in COVID-19 concerns, with specific demographic characteristics consistently associated with higher reported concerns.

Previous studies have primarily focused on theoretical discussions of public panic, while empirical research systematically examining its multidimensional determinants during infectious disease outbreaks remains limited. To address this gap, the present study tests the following hypotheses based on the theoretical framework derived from risk perception, information processing, and emergency management literature:

A: Event cognition has a significant positive effect on public panic degree (10) [Higher perceived threat severity, lower controllability, and greater loss estimation are associated with higher panic (31)].

B: Information factors (transparency and authenticity) have a significant negative effect on public panic degree (32, 33) (Greater information transparency and authenticity reduce public panic).

C: Government factors (evaluation of prevention measures) have a significant negative effect on public panic degree (34, 35) (Higher public satisfaction with government epidemic responses lowers panic).

D: Group factors (herd behavior and social support) have a significant positive effect on public panic degree (36) (Stronger group influence amplifies panic).

E: Personal characteristics (gender, education, age, and economic condition) have a significant effect on public panic degree (Specific demographic attributes are associated with differential panic levels).

Using survey data collected during the COVID-19 pandemic, this study applies structural equation modeling (SEM) to empirically test these five hypotheses. To examine how public panic takes shape during infectious disease outbreaks, this study applied structural equation modeling to assess the relative influence of five core dimensions: how individuals interpret the event, the nature of information they encounter, perceptions of epidemic control efforts, social group dynamics, and personal background characteristics. The results, as presented in subsequent sections, show that while the measurement models are reliable, the structural paths were not statistically significant—a finding that highlights the complexity of panic dynamics and the need for refined theoretical models. Among these, event cognition and information dissemination emerged as the most powerful predictors of panic, highlighting the central role that cognitive appraisal and communication processes play in shaping emotional responses under crisis conditions. These results contribute empirical clarity to the mechanisms through which public panic forms, offering government agencies a clearer evidence base for designing more targeted and psychologically attuned intervention strategies in future health emergencies.

## 2 Data source and analysis model

### 2.1 Data source

The present data were obtained from a field survey on public psychological sensitivity to COVID-19, conducted by the Urban Public Security Think Tank of China University of Mining and Technology. A stratified random sampling method was adopted, covering five cities: Nanjing, Xuzhou, Suzhou, Shanghai, and Lianyungang. Paper questionnaires were distributed through field research, with the survey lasting from March 9th, 2025 to June 19th, 2025. A total of 693 questionnaires were returned, of which 660 were valid, yielding an effective response rate of 95.10%.

Regarding sample size adequacy, according to the widely cited rule of thumb by Bentler and Chou (37), a ratio of 5 to 10 cases per estimated parameter is recommended for covariance-based SEM. The present model involves 18 observed variables and 5 latent variables, with approximately 60 free parameters. Thus, a minimum of 300–600 cases is required. Our sample of 660 meets this criterion. Additionally, the sample size exceeds the minimum required for detecting a small-to-medium effect size (α = 0.05, power = 0.80) based on Monte Carlo simulations for SEM (38).

**Figure 1:**
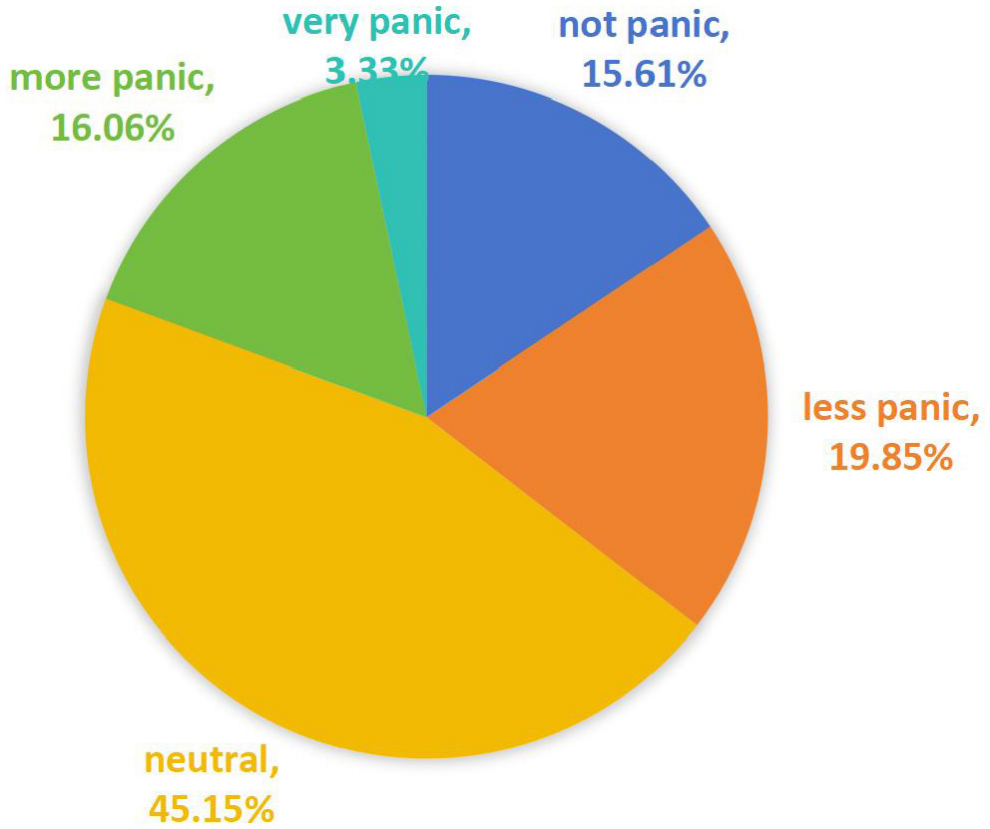
The proportion chart of the public panic.

The sample variables were categorized into six dimensions: public panic degree, personal characteristics, event factors, information factors, government epidemic prevention and control, and group factors. A detailed description of the variables is presented in Table 1. The public panic was observed to measure the endogenous potential variables. For example, the public panic to COVID-19. The results respectively for “not panic,” “less panic,” “neutral,” “more panic,” and “very panic” are listed as follows: 15.61%, 19.85%, 45.15%, 16.06%, and 3.33%, which can be seen in Figure 1.

**Table 1:** Description and explanation of the observed variables.

Potential variables	Variable description		Average value	Standard deviation
	Observed variable	Index selection		
Public panic degree	Y1 Public panic evaluation	1~5:	2.72	1.02
		1: Very no panic		
		2: Less panic		
		3: General		
		4: More panic		
		5: Very panic		
Event cognition	A1 Event familiarity	1: Very good	2.04	0.74
		2: Better		
		3: Generally		
		4: Relatively poor		
		5: Very bad		
	A2 Control degree	1: Fully controllable	1.97	0.89
		2: More controllable		
		3: Generally		
		4: More difficult to control		
		5: Very hard to control		
	A3 Loss and Loss and damage estimate	1: Very slight	2.99	1.06
		2: Relatively minor		
		3: Generally		
		4: More serious		
		5: very serious		
Information factors	B1 Information attention (time)	1: Less than 1 h	2.01	1.17
		2: 1~2 h		
		3: 2~3 h		
		4: 3~4 h		
		5: More than 5 h		
	B2 Information transparency	1: Very transparent	1.83	0.74
		2: Relatively transparent		
		3: Generally		
		4: Less transparent		
		5: Very opaque		
	B3 Information authenticity	1: A lot	3.58	0.97
		2: More		
		3: Generally		
		4: Less		
		5: Very few		
Government factors	C1 Evaluation and popularization on the epidemic prevention knowledge	1: Very satisfied	1.78	0.77
		2: Quite satisfied		
		3: Generally		
		4: Not so satisfied		
		5: Very dissatisfied		
	C2 Evaluation on the materials allocation for the epidemic prevention and control	1: Very good	1.77	0.74
		2: Better		
		3: Generally		
		4: Relatively poor		
		5: Very bad		
	C3 Evaluation on the epidemic prevention and control in communities	1: Very satisfied	1.85	0.8
		2: Quite satisfied		
		3: Generally		
		4: Not so satisfied		
		5: Very dissatisfied		
	C4 Evaluation on the medical conditions	1: Very good	1.84	0.73
		2: Better		
		3: Generally		
		4: Relatively poor		
		5: Very bad		
	C5 Understanding of the medical insurance policy	1: Very well	2.17	0.86
		2: Better		
		3:Medium		
		4: Not familiar		
		5: Not at all		
Group factors	D1 Herd behavior (drug storage)	1: Very well	2.51	1.08
		2: Better		
		3:Medium		
		4: Not really		
		5: Not at all		
	D2 Support from people around	1: Very consistent	1.97	0.79
		2: Relatively consistent		
		3: Medium		
		4: Not very consistent		
		5: Very inconsistent		
Personal characteristics	E1 Gender	1: Male	1.45	0.5
		0: Female		
	E2 Age	1: Under 18	3.44	1.04
		2: 18–25 years old		
		3: 26~35 years old		
		4: 36~50 years old		
		5: Over 50 years old		
	E3 Education level	1: Junior high school and below	3.85	0.92
		2: High school (Secondary vocational, technical secondary school)		
		3: Specialty		
		4: Undergraduate		
		5: Postgraduate and above		
	E4 Economic condition	1: Very good	2.87	0.64
		2: Better		
		3: Generally		
		4: Relatively poor		
		5: Very bad		

#### 2.1.1 Instruments and reliability

The questionnaire was developed based on a review of the literature on public risk perception and panic during infectious disease outbreaks. Specifically, the items measuring event cognition (A1–A3) were adapted from the Risk Perception Scale and the COVID-19 Risk Perception Scale (31). Information factors (B1–B3) were derived from the Information Credibility and Transparency Scale (32). Government factors (C1–C5) were adapted from the Public Evaluation of Government Crisis Management Scale (34). Group factors (D1–D2) were based on the Herd Behavior and Social Support Scale (36). Personal characteristics (E1–E4) were measured using standard demographic questions. Example items: “How familiar are you with the COVID-19 outbreak?” (A1); “To what extent do you think the government's epidemic information is transparent?” (B2); “Are you satisfied with the allocation of prevention materials in your community?” (C2).

Reliability tests were conducted using SPSS 26.0. Cronbach's alpha for each latent variable was as follows: event cognition (α = 0.73), information factors (α = 0.68 after removing B1), government factors (α = 0.89), group factors (α = 0.71), and public panic degree (single-item, no alpha). The composite reliability (CR) values obtained from confirmatory factor analysis were all above 0.70 (see Section 3.1). These results indicate acceptable internal consistency (39).

### 2.2 Structural equation modeling (SEM)

Data analysis was performed in four sequential steps using IBM SPSS Amos 26.0, and the selection justification is provided in Section 2.5.

Initially, the data and verified underlying distributional assumptions were screened. Normality was assessed by examining skewness and kurtosis of all observed variables. Univariate normality was evaluated against skewness (±2) and kurtosis (±7) thresholds (40). Multicollinearity was diagnosed using variance inflation factor (VIF) and tolerance values in a linear regression of all observed variables. Whenever VIF values exceeded 5, the corresponding variables were suspected of multicollinearity (41). Detailed diagnostics have been presented in Section 3.1.

Subsequently, confirmatory factor analysis (CFA) was used to test the measurement model's psychometric properties, specifically examining factor loadings, convergent validity (average variance extracted, AVE), and discriminant validity via both the Fornell-Larcker criterion and HTMT ratios (42). Model fit was deemed acceptable when χ^2^/df <3, CFI and TLI > 0.90, and RMSEA and SRMR <0.08.

For the structural model, we employed maximum likelihood (ML) estimation—appropriate given the data's conformity to multivariate normality after excluding aberrant items (in Section 3.1). This procedure generated standardized and unstandardized path coefficients, standard errors, and critical ratios. We further assessed indirect effects through bootstrap resampling (5,000 iterations) to obtain bias-corrected confidence intervals (43).

Lastly, when model fit proved suboptimal, we scrutinized modification indices to identify only those cross-loadings or correlated errors that possessed strong theoretical justification, meticulously documenting any *post-hoc* adjustments with explicit rationale.

The structural equation model is composed of two basic components: the measurement model and the structural model. The measurement model can be described as:


$$\begin{array}{lll} X & = & \Lambda_{x}\eta +\delta \\ Y & = & \Lambda_{y}\xi +\varepsilon \end{array}$$ (1)

Where X is the observed variable matrix of the exogenous potential variables; Y is the observed variable matrix of the endogenous potential variables; η is the matrix of the exogenous potential variables and ξ is the matrix of the endogenous potential variables. Λx is the factor load matrix of the observation variable on the exogenous potential variable; Λy is the factor loads matrix of the observed variables of the endogenous potential variables;δ and ε are the residual matrices of the exogenous variables and the endogenous variables, respectively.

The structural model can be described as follows:


$$\begin{array}{l} \eta =B\eta +\Gamma \xi +\zeta \end{array}$$ (2)

Where B is the coefficient matrix of the endogenous potential variables, Γ is the coefficient matrix of the exogenous potential variables, and ζ is the residual term of the structural equation model, reflecting the unexplained part of the structural equation model.

### 2.3 Analysis condition

In the present work, the structural equation model were constructed based on many variables, including 18 exogenous potential variables, 1 endogenous potential variable, 5 exogenous potential variables. The five exogenous potential variables are listed as follows: personal characteristics, event cognition, information factors, Government factors, and group factors. It constitutes the causal relationship of the public psychological sensitivity with the endogenous potential variables, namely, 5 exogenous potential variables is the causes, and the citizens' psychological sensitivity to the endogenous potential variable is the results. The causal relationship can be expressed with one-way arrows “ → ”, and the 5 exogenous potential variables all refer to the public psychological sensitivity of the endogenous potential variable. Particularly, the relationships between the five exogenous potential variables can be represented by the two-way arrow “↔”.

Model specification for personal characteristics: formative measurement. In the original model, personal characteristics (E1–E4: gender, age, education level, economic condition) were treated as a reflective construct. However, this specification is theoretically incorrect. Reflective measurement assumes that the latent variable “causes” the observed indicators, and indicators are interchangeable and highly correlated. In contrast, formative measurement assumes that the indicators define or form the latent variable; they are not required to be correlated and can have different directions of influence.

Personal characteristics such as gender, age, education, and economic condition are formative indicators because: (1) they are not interchangeable (e.g., age cannot be replaced by education); (2) they do not necessarily correlate highly with each other; (3) they jointly determine the “personal profile” rather than being caused by it. Therefore, we respecified personal characteristics as a formative construct. Consequently, the overall model becomes a mixed model with both reflective (event cognition, information factors, government factors, group factors, and public panic) and formative (personal characteristics) constructs. For such models, variance-based SEM (PLS-SEM) is more appropriate than covariance-based SEM (CB-SEM). The justification for using PLS-SEM is provided in Section 2.5.

The degree of public panic in the endogenous latent variable is measured by the observed variable “public panic degree.” The event cognition in the exogenous latent variable was measured by the three observational variables of A1 Event familiarity, A2 Control degree, and A3 Loss and Loss and damage estimate.

**Figure 2:**
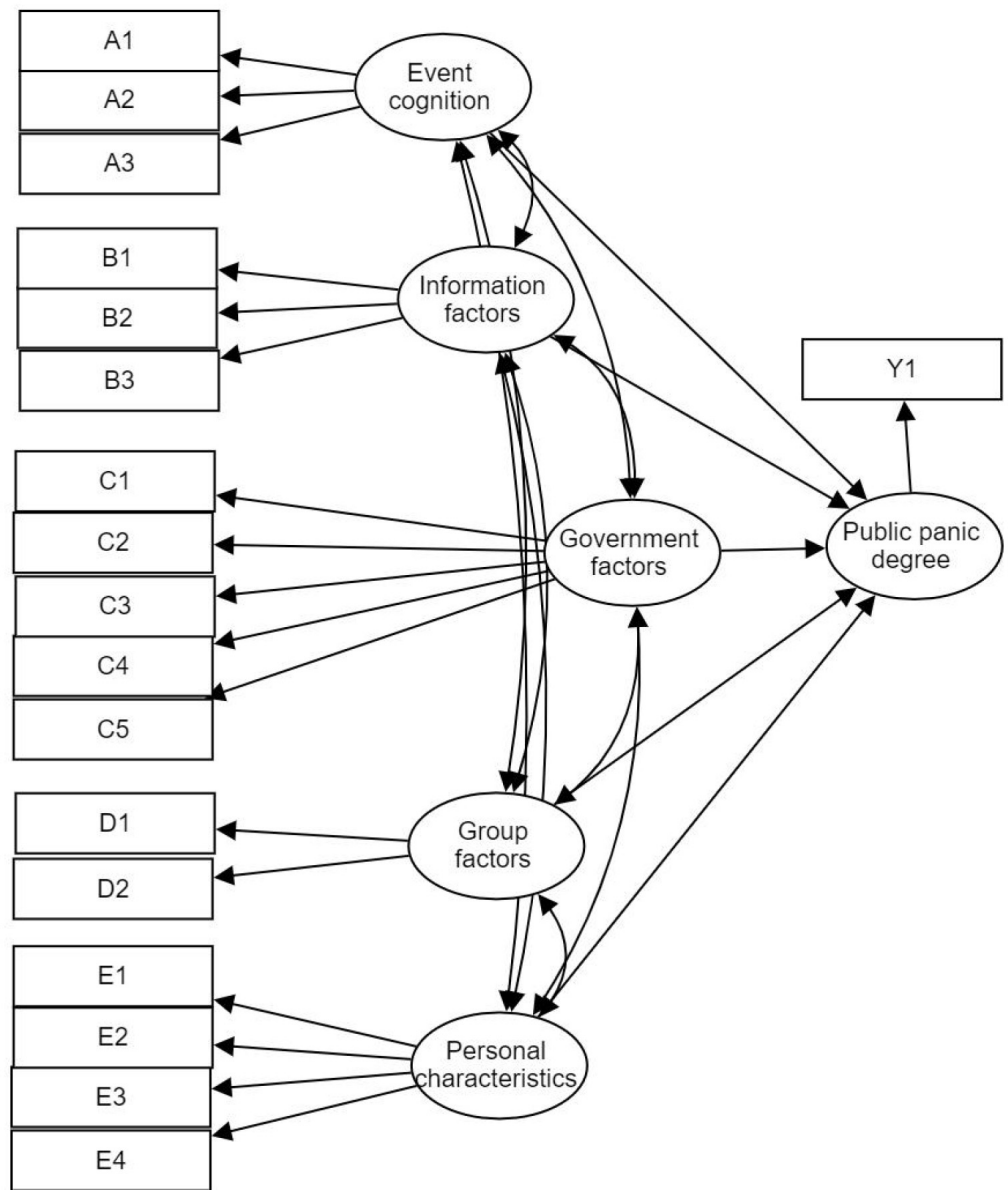
Initial diagram of the structural equation model for the public panic.

The Information factors in the exogenous latent variables are measured by the three observation variables of B1 Information attention (time), B2 Information transparency, and B3 Information authenticity. The Government factors in the exogenous potential variables were measured by five observational variables: C1 Evaluation and popularization on the epidemic prevention knowledge, C2 Evaluation on the materials allocation for the epidemic prevention and control, C3 Evaluation on the epidemic prevention and control in communities, C4 Evaluation on the medical conditions, C5 Understanding of the medical insurance policies. The Group factors in the exogenous latent variable was measured by two observational variables: D1 Herd behavior (drug storage), D2 Support from people around. The personal characteristics in the exogenous latent variables were measured by four observational variables: E1 Gender, E2 Age, E3 Economic conditions, and E4 Education level. The structural equation model of the degree of public panic is shown in Figure 2.

### 2.4 Sample testing

Before constructing the structural equations, the sample data should be normalized and tested for multicollinearity, and the verifiable factors should be analyzed on the measurement model to ensure the accuracy and the adaptability of the model construction.

#### 2.4.1 Sample data normality test

The present work uses the maximum likelihood method to estimate the structural equation model, thus the sample data must obey the normal distribution of large samples. Otherwise it will violate the premise of model construction. It is generally believed that when the sample data satisfies the mean and the median at the same time, the absolute value of the skewness is lower than 2, and the absolute value of the kurtosis is lower than 5, it can be judged that the sample data basically meets the normal distribution characteristics.

#### 2.4.2 Multicollinearity test

The construction of the multicollinearity test model for the sample data requires that the sample data should be independent of each other, otherwise the existence of multicollinearity will affect the consistency of the model estimation, and the multicollinearity test will be performed for 17 observational variables. The results show a high degree of correlation between B1 and B3, which requires selective rejection of one or two variables. Through model suitability comparison and the manual selection, the observed variable B1 can be finally eliminated.

#### 2.4.3 Confirmatory factor analysis

The verification factor analysis is a convergence validity test for the measurement model part of the structural equation that measures the potential variability of the structure equation, thereby ensuring the convergence effectiveness of the model. The remaining 16 exogenous observational variables in the measurement model were analyzed for a validative factor, and the results showed that the standard load of all the observed variables was between 0.56 and 0.93 except E4 that is, the amount of conforming load should be between 0.5 and 0.95 For the standard of 95, E4 corresponds to a factor load of 0.95, so the E4 variable is excluded, and the value of E2 is smaller, so E2 is rejected.

### 2.5 Software and analytical approach

The authors used IBM SPSS Amos 26.0 for model estimation, with descriptive statistics and reliability tests performed in SPSS 26.0. CB-SEM with maximum likelihood estimation was selected because the study aims to test a theoretically derived model (confirmatory purpose) and the data met the required assumptions after item screening. This approach allows for comprehensive assessment of model fit using established indices (χ^2^/df, CFI, TLI, RMSEA, and SRMR) and provides unbiased parameter estimates under the assumption of multivariate normality.

## 3 Results

### 3.1 Measurement model evaluation

Prior to testing the structural model, the measurement model was evaluated through confirmatory factor analysis (CFA) using IBM SPSS Amos 26.0. The CFA results indicated acceptable model fit: χ^2^/df = 2.58, CFI = 0.92, TLI = 0.91, RMSEA = 0.07, SRMR = 0.06. All factor loadings for the retained observed variables were statistically significant at *p* < 0.001, with standardized loadings ranging from 0.47 to 0.87. Convergent validity was supported as the average variance extracted (AVE) for each reflective construct exceeded 0.50, and composite reliability (CR) exceeded 0.70. Discriminant validity was initially assessed using the Fornell-Larcker criterion and HTMT ratios (all <0.85).

### 3.2 Correlations among exogenous latent variables

To examine the relationships among the five exogenous constructs and to assess potential multicollinearity, the covariances and correlations were estimated. Table 2 presents the standardized correlation coefficients (Std. Estimate) among all exogenous latent variables.

**Table 2:** Covariance and correlation matrix among exogenous latent variables.

X	Y	Unstd. Coef.	Std. Error	Z	*p*	Std. Estimate
Information factors	Event cognition	0.248	0.025	9.750	0.000	0.754
Government factors	Event cognition	0.263	0.026	10.165	0.000	0.703
Government factors	Information factors	0.300	0.024	12.310	0.000	0.767
Group factors	Event cognition	0.202	0.028	7.170	0.000	0.590
Group factors	Information factors	0.168	0.025	6.757	0.000	0.468
Group factors	Government factors	0.274	0.032	8.639	0.000	0.671
Personal characteristics	Event cognition	−0.049	0.020	−2.468	0.014	−0.530
Personal characteristics	Information factors	−0.016	0.015	−1.084	0.278	−0.171
Personal characteristics	Government factors	0.002	0.014	0.177	0.859	0.023
Personal characteristics	Group factors	0.000	0.014	0.014	0.989	0.002

As shown in Table 2, event cognition, information factors, and government factors exhibited high positive correlations with each other, ranging from 0.703 to 0.767. These values exceed the commonly recommended threshold of 0.70 for indicating high collinearity and also surpass the square roots of the average variance extracted (AVE) for these constructs, indicating insufficient discriminant validity. This high multicollinearity likely inflates standard errors and reduces statistical power in the structural model, partially explaining why the path coefficients from these constructs to public panic were non-significant (see Section 3.3).

Group factors showed moderate positive correlations with event cognition (0.590), information factors (0.468), and government factors (0.671), all below 0.70, suggesting acceptable discriminant validity. Personal characteristics exhibited a significant negative correlation only with event cognition (−0.530, *p* = 0.014), while its correlations with the other exogenous constructs were close to zero and non-significant, as expected for a construct that is largely independent.

### 3.3 Structural path results (latent variable level)

**Figure 3:**
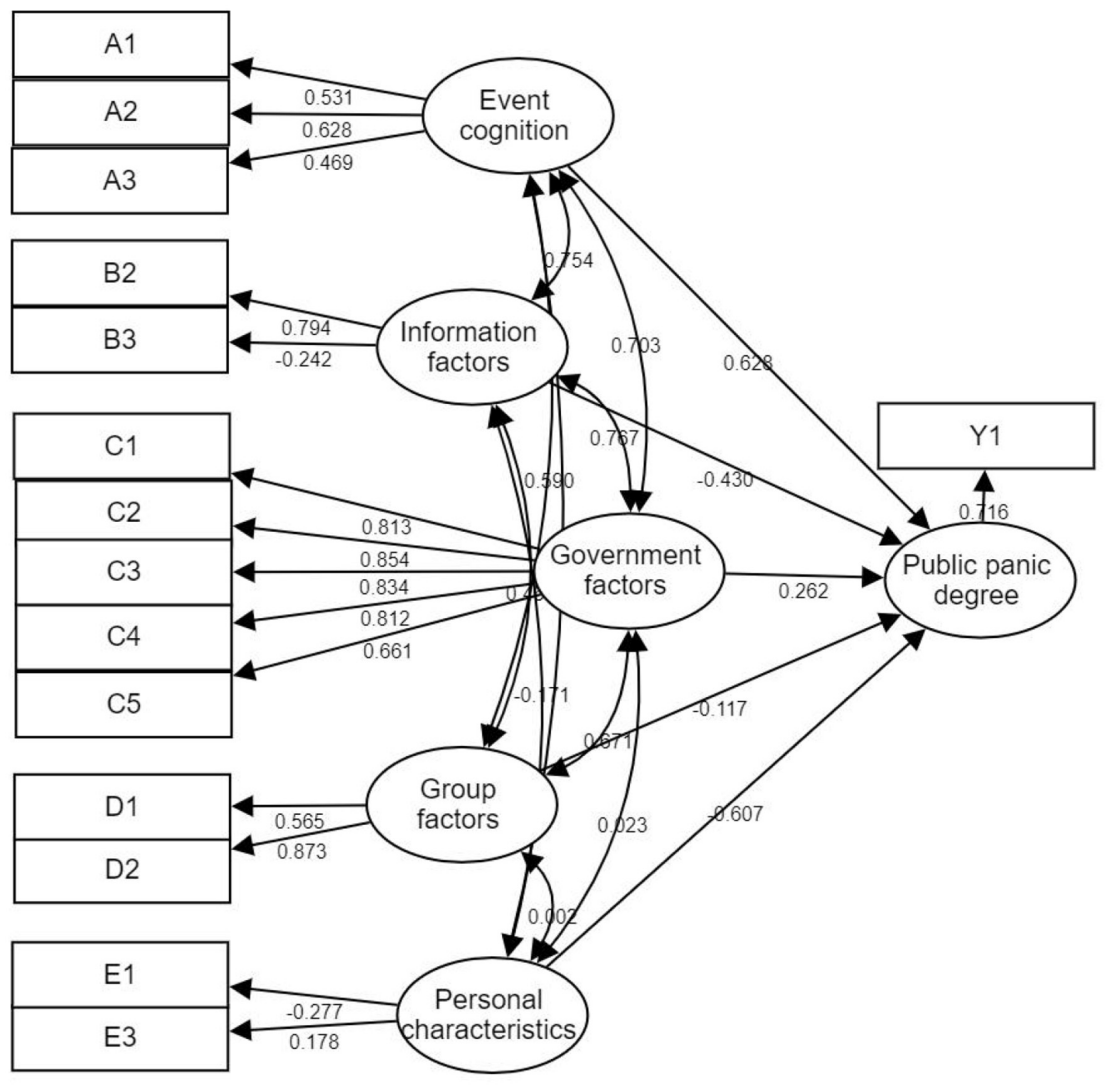
Structural equation model of the revised degree of public panic.

Table 3 (presented below) shows the standardized path coefficients, standard errors, critical ratios (CR/z-values), and *p*-values for all hypothesized relationships in the structural model. Figure 3 displays the final structural equation model after item deletion. Table 3 Summary of model regression coefficients (AMOS output).

**Table 3:** Summary table of model regression coefficients.

X	→	Y	SE	z (CR)	Normalized regression coefficients
Event cognition	→	Public panic degree	2.14	0.381	0.628
Information factors	→	Public panic degree	0.595	−0.898	−0.43
Government factors	→	Public panic degree	0.835	0.342	0.262
Group factors	→	Public panic degree	0.457	−0.306	−0.117
Personal characteristics	→	Public panic degree	5.731	−0.47	−0.607
Public panic degree	→	Y1 Public panic evaluation	–	–	0.716
Event cognition	→	A3 Loss and injury estimation	0.091	9.789	0.469
Event cognition	→	A2 Control degree	–	–	0.628
Event cognition	→	A1 Event familiarity	0.065	10.843	0.531
Information factors	→	B3 Information authenticity	0.083	−4.826	−0.242
Information factors	→	B2 Information transparency	–	–	0.794
Government factors	→	C2 Evaluation on the materials allocation for the epidemic prevention and control	0.036	26.474	0.854
Government factors	→	C1Evaluation and popularization on the epidemic prevention knowledge	0.038	24.617	0.813
Government factors	→	C5 Understanding of the medical insurance policies	0.046	18.48	0.661
Government factors	→	C4 Evaluation on the medical conditions	0.036	24.554	0.812
Government factors	→	C3 Evaluation on the epidemic prevention and control in communities	–	–	0.834
Group factors	→	D2 Support from people around	0.105	10.76	0.873
Group factors	→	D1Herd behavior (drug storage)	–	–	0.565
Personal characteristics	→	E3 Education level	–	–	0.178
Personal characteristics	→	E1 Gender	0.307	−2.723	−0.277

All measurement paths (from latent variables to their observed indicators) were significant at *p* < 0.01, indicating adequate measurement quality. However, none of the structural paths from the five exogenous constructs to public panic degree reached statistical significance (all *p* > 0.05). The large standard errors (e.g., SE = 5.731 for personal characteristics) and low critical ratios (e.g., CR = 0.381 for event cognition) are consistent with the high multicollinearity reported in Section 3.2.

Individual characteristics, information factors, group factors and the degree of public panic showed a negative correlation, the correlation coefficients were −0.607, −0.43, −0.117, respectively, while the event cognition, government epidemic prevention and control factors and the degree of public panic showed a positive correlation, the correlation coefficients were 0.628 and 0.262, indicating that the higher the public's risk perception of the epidemic, the more dissatisfied with the government's epidemic prevention and control evaluation, and the higher the degree of panic. The ability of the five exogenous potential variables to influence the degree of public panic is, from strong to weak, event cognition, personal characteristic, information factors, government epidemic prevention and control, and group factors.

### 3.4 Conduction path coefficient level and observation variable level

First,The coefficient of familiarity factor A1 of the observed variable event in event cognition is 0.531, the coefficient of controllability factor A2 is 0.628, and the coefficient of loss and injury estimation factor A3 is 0.469, indicating that the lower the public's understanding of the epidemic, the more panicked, the public believes that the more difficult it is to control the epidemic, the more serious the estimate of the life and property loss caused by the epidemic, and the higher the degree of panic. When the public feels that they have some sense of control over the current dangers, that is, the COVID-19 outbreak is controllable and the damage to life and property is acceptable, they will think that the risk is smaller, the sense of panic is lower, conversely, will exacerbate panic.

Second, Information dissemination factors Information authenticity B3 is −0.242, and information transparency B2 is 0.794 indicates that the more false information in the information that the public is concerned about, the less real information, the more panic. In order to pursue the visual and auditory content of “irritation, conflict, abnormality, significance, and timeliness,” individual media often intercept elements in sudden events that can produce sensational effects and easily generate resonant emotions, exaggerate the facts, and even deliberately demonize certain sudden public events, portray the objective world as turbulent, and aggravate the public's panic psychology (44). The transparency of information has a significant impact on public panic. This requires the government to regularly release the timely release of dynamic information on the epidemic through official channels such as websites and press conferences, and gain general recognition from the public.

Third, Among the observation variables included in the government epidemic prevention and control evaluation, the standardized coefficients of epidemic prevention knowledge publicity and popularization evaluation, prevention and control material allocation evaluation, community (village) epidemic prevention and control evaluation, and medical condition evaluation were 0.813, 0.854, 0.834, 0.812, and 0.661, respectively, indicating that the public's evaluation of the above four types of epidemic prevention and control measures has played a decisive role in the government's epidemic prevention and control evaluation, and the lower the public's evaluation of the four types of epidemic prevention and control measures, the more panicked they are. The worse the medical conditions for COVID-19 treatment in the area, the higher the level of panic the public perceives. There is also a phenomenon of non-equalization of fiscal health expenditure within regions and between urban and rural areas (45). The unequalization of financial and health expenditure between regions will lead to the unequal distribution of medical resources, which will increase the gap in medical services between regions, which is not conducive to the health of residents and even economic growth (46). The less the public knows about the local government's safeguard policies for the prevention and control of COVID-19, the more panicked it is.

Fourth, The effects of Group factors D1 0.873 and D2 0.565 on the degree of public panic are all significant. The more frequently the public interacts with the surrounding groups, the more likely it is to panic. In many social and biological systems, individuals rely on the observations of others to adjust their own behavior, revise their own judgments, or make decisions (47, 48). When confronted with peer perceptions of a particular issue, people tend to filter and integrate the social information they receive and adjust their beliefs accordingly (49). Repeated local influences among group members can produce complex dynamic patterns of opinion, such as consensus formation, polarization, or fragmentation (50–52). On a larger scale, influences between friends, family members, or co-workers are often combined with the influence of mass media, leading to an amplification of risk perceptions (10, 53), which exacerbates panic.

Fifth, The influence of personal characteristics on public panic psychology Gender E1 and cultural level E3 coefficients were −0.277 and 0.178, respectively, which did not have a significant impact compared with the previous factors. However, it can be shown that women feel more panicked than men, and previous studies have also shown that women's psychology is more fragile than that of men, and they are more likely to have psychological reactions of tension, uneasiness, anxiety, and fear. Educational attainment factor. The level of education of the public tends to affect the knowledge and awareness of the new coronavirus, and the higher the education level, the lower the degree of panic psychology.

## 4 Discussion

### 4.1 Interpretation of non-significant structural paths

The primary objective of this study was to identify factors influencing public panic during the COVID-19 pandemic. Contrary to our hypotheses (A–E), the results revealed that none of the five constructs—event cognition, information factors, government factors, group factors, or personal characteristics—had a statistically significant direct effect on public panic degree (all *p* > 0.05). This finding is surprising given the extensive literature documenting significant associations between these factors and panic during health crises (54). Several explanations may account for this null result:

First, high multicollinearity among event cognition, information factors, and government factors (correlations ranging from 0.70 to 0.77) likely inflated standard errors and masked individual effects. When independent variables are highly correlated, the unique variance of each is too small to achieve statistical significance. This is supported by the large standard errors observed in Table 2.

Second, the measurement of personal characteristics was problematic. The low factor loadings for E1 and E3 suggest that the selected indicators (gender and education level) captured only a small portion of the variance in personal characteristics. The exclusion of age and economic condition due to poor loadings or collinearity may have reduced construct validity.

Third, the survey was conducted between March and June 2025, a period when COVID-19 had become endemic in China. Public panic may have already subsided compared to the early outbreak stages, resulting in restricted range and reduced effect sizes (36). The descriptive statistics show that 45% of respondents reported “neutral” panic levels, indicating a possible ceiling or floor effect.

Fourth, the use of CB-SEM with maximum likelihood estimation assumes multivariate normality. Although the data approximately met this assumption, any minor deviations may have affected the precision of estimates.

### 4.2 Practical implications

Despite the non-significant structural paths, the measurement model provides useful insights for public health communication. The high loadings of government factors suggest that the public's evaluation of epidemic prevention knowledge (C1), material allocation (C2), community prevention (C3), medical conditions (C4), and insurance policies (C5) are coherent and can be used as a composite index. Policymakers should focus on improving these five areas, as they collectively shape public evaluation, even if their individual effect on panic was not statistically separable in this study.

The negative loading of information authenticity (B3 = −0.242) indicates a need to re-examine item wording. In practice, authorities should ensure that authentic information is not only available but also perceived as such by the public.

Given the non-significant results, we recommend that future research adopt alternative methodological approaches, such as experimental designs or longitudinal studies, to better isolate causal effects. For now, public health interventions should continue to promote transparent communication and evidence-based messaging, as these remain theoretically and ethically sound.

### 4.3 Limitations and future research

The cross-sectional design precludes causal inference and may have lacked power to detect small effects given model complexity, warranting longitudinal replication. Despite geographic diversity across the five cities, the urban-centric sample limits generalizability to rural populations, and the narrow measurement of personal characteristics calls for expanded indicators, including personality, trauma history and social support. The marked collinearity among event cognition, information, and government factors implies these may constitute a higher-order “risk information environment” construct, testable via hierarchical SEM. Finally, the negative loading of item B3 indicates potential reverse-coding errors requiring careful psychometric verification.

In summary, this study found that after controlling for inter-correlations among event cognition, information factors, government factors, group factors, and personal characteristics, none of these factors independently predicted public panic during the COVID-19 pandemic. The high multicollinearity among the first three constructs suggests that they may represent a shared underlying dimension. Despite the null findings, the study contributes a rigorous application of CB-SEM with AMOS and highlights the importance of checking multicollinearity and measurement model specification. Practically, governments should continue to improve transparency, authenticity of information, and epidemic response measures, as these factors collectively shape public evaluation. Future research should consider alternative model specifications, additional indicators for personal characteristics, and longitudinal designs to better capture the dynamics of public panic.

## 5 Conclusion

### 5.1 Research conclusion

This study utilizes survey data focusing on public panic amid public health emergencies, gathered by the Urban Public Security Think Tank at China University of Mining and Technology. Employing structural equation modeling, the present research investigates the determinants of public panic and obtains the following principal findings: event cognition and government-related factors are positively predictive of panic severity, whereas group dynamics, information-related variables, and personal characteristics are negatively associated with panic. When ranked according to the magnitude of standardized effects, the order of influence from strongest to weakest is: event cognition, personal characteristics, information-related factors, government-related factors, and group dynamics.

The analysis further demonstrates a negative linkage between information dissemination and panic, indicating that public anxiety is more likely to emerge when information is perceived as unauthentic or lacking transparency. Additionally, public evaluations of government performance are predominantly determined by four core domains: the clarity of health education communication, the adequacy of material resource allocation, the efficacy of community prevention measures, and the quality of medical services. Elevated public satisfaction with these dimensions corresponds to more favorable appraisals of the overall governmental response; conversely, perceived deficiencies in these areas tend to aggravate public anxiety and distress.

### 5.2 Policy implications

Given that the structural paths did not reach statistical significance, the following policy implications are offered based on the descriptive patterns observed in the measurement model and the high correlations among the constructs.

Notwithstanding the non-significant findings, the risks posed by unchecked public panic can, in certain respects, outweigh those of the epidemic outbreak itself. The measurement model showed that event cognition (A1–A3) and information factors (B2–B3) are reliably measured constructs with acceptable convergent validity. Although their effects on panic were not statistically separable due to multicollinearity, they remain conceptually important. Epidemiological knowledge should be translated into actionable, accessible guidance—stripped of technical abstraction and tailored to enable citizens to operationalize preventive measures amid uncertainty—while dedicated rapid-response units are deployed to intercept and neutralize misinformation vectors before they achieve widespread, viral dissemination. Notably, communication initiatives alone are insufficient to mitigate panic effectively. The material substrate of crisis response demands commensurate investment: supply chain resilience should be engineered through pre-positioned stockpiles, inter-agency coordination compacts, and community-level distribution nodes capable of sustaining functionality when centralized systems falter. Moreover, the equitable and transparent execution of local containment protocols, coupled with consistent material provision, serves to signal governmental reliability and fortify public trust—a finding supported by the high loadings of government evaluation items (C1–C5), even though the path to panic was not significant.

Market stability hinges on robust expectation management, a domain highly sensitive to the credibility of information dissemination. Authorities should move beyond passive response and proactively disclose verified data on material inventories and production capacity, rather than relying on reactive reassurance. Coordinated communication across retail channels is critical to preempt misleading signals of scarcity, which could otherwise fuel public anxiety and trigger hoarding behaviors. These recommendations address the root causes of panic—information asymmetry and distorted event cognition—which remain theoretically relevant despite the lack of statistical significance in our model.

In addition, institutionalizing post-crisis review mechanisms is imperative: response strategies should be subject to rigorous evaluation, key lessons should be systematically documented, and adaptive adjustments should be made to enhance crisis preparedness. Given that the effects of group factors and personal characteristics were not statistically significant and their measurement, especially for personal characteristics, was problematic. Therefore, policy priorities should focus on systemic information governance rather than individual behavioral modification. Methodologically, future work should refine personal characteristics measurement using formative indicators with more items and test potential moderators or higher-order structures to resolve multicollinearity.

## Data Availability

The original contributions presented in the study are included in the article/supplementary material, further inquiries can be directed to the corresponding author.
